# Adaptation of the Compliance with Standard Precautions Scale for Nurses in Clinical Settings (Japanese Version): A Two-Phase Methodological Study

**DOI:** 10.14789/ejmj.JMJ25-0054-OA

**Published:** 2026-04-10

**Authors:** KANA TAMURA, HIROAKI ITOH, ASAKO YASUNO, SIMON CHING LAM, SAKIKO IGAWA, KEIKO OSHIMA, AKIHIKO SHIMIZU, YOKO BUYA, YUKARI TAKAI, EMIKO UBUKAWA, KAZUHITO YOKOYAMA

**Affiliations:** 1 Department of Epidemiology and Environmental Health, Graduate School of Medicine, Juntendo University, Tokyo, Japan; 1 Department of Epidemiology and Environmental Health, Graduate School of Medicine, Juntendo University, Tokyo, Japan; 2 Faculty of Nursing, Jobu University, Gunma, Japan; 2 Faculty of Nursing, Jobu University, Gunma, Japan; 3 Department of Epidemiology and Environmental Health, Juntendo University Faculty of Medicine, Tokyo, Japan; 3 Department of Epidemiology and Environmental Health, Juntendo University Faculty of Medicine, Tokyo, Japan; 4 Faculty of Health Care Department of Nursing, Takasaki University of Health and Welfare, Gunma, Japan; 4 Faculty of Health Care Department of Nursing, Takasaki University of Health and Welfare, Gunma, Japan; 5 School of Nursing, Tung Wah College, Hong Kong SAR, China; 5 School of Nursing, Tung Wah College, Hong Kong SAR, China; 6 Infection Control Nurse, Takasaki University of Health and Welfare, Gunma, Japan; 6 Infection Control Nurse, Takasaki University of Health and Welfare, Gunma, Japan; 7 Chief Infection Control Nurse, Department of Nursing, Gunma University Hospital, Gunma, Japan; 7 Chief Infection Control Nurse, Department of Nursing, Gunma University Hospital, Gunma, Japan; 8 Infection Control Doctor, Gunma Children’s Medical Center, Gunma, Japan; 8 Infection Control Doctor, Gunma Children’s Medical Center, Gunma, Japan; 9 Infection Control Microbiological Technologist, JCHO Gunma Central hospital, Gunma, Japan; 9 Infection Control Microbiological Technologist, JCHO Gunma Central hospital, Gunma, Japan; 10 Department of Nursing, School of Nursing, Gunma Prefectural College of Health Sciences, Gunma, Japan; 10 Department of Nursing, School of Nursing, Gunma Prefectural College of Health Sciences, Gunma, Japan; 11 Department of Epidemiology and Social Medicine, International University of Health and Welfare Graduate School of Public Health, Tokyo, Japan; 11 Department of Epidemiology and Social Medicine, International University of Health and Welfare Graduate School of Public Health, Tokyo, Japan

**Keywords:** adherence, compliance, healthcare associated infections, nursing, standard precautions

## Abstract

**Objectives:**

To translate and adapt the Compliance with Standard Precautions Scale (CSPS) into Japanese and validate it among Japanese nurses.

**Materials and Methods:**

This was a two-phase study. First, we translated the original English CSPS into Japanese. The content validity index of the result was evaluated by experts, whereas the linguistic validity was ascertained by clinical nurses, from which we generated the CSPS-Japanese version. Second, the reliability and validity of the CSPS-Japanese version were evaluated using cross-sectional data from 248 nurses. Reliability was assessed using internal consistency (Cronbach’s alpha) and the 2-week test-retest reliability (intraclass correlation coefficient). Construct validity was established using confirmatory factors and Rasch analysis. Differences in self-reported compliance based on training experience were examined.

**Results:**

The CSPS-Japanese version showed satisfactory reliability. The constructed model showed an acceptable fit index. Nurses with training experience had higher self-reported compliance.

**Conclusions:**

The CSPS-Japanese version showed good reliability and validity, and this indicates that the tool constitutes an appropriate new self-administered evaluation method for Japanese nurses.

## Introduction

Standard precautions, introduced by the CDC in 1996 and emphasized by the WHO, are foundational to infection prevention in healthcare settings. These measures—including hand hygiene and the use of Personal Protective Equipment (PPE)—are universally applied to reduce risks from blood and bodily fluids, regardless of infection status^1)^. The WHO also emphasizes their universal application, regardless of infection status, to protect both healthcare workers and patients^2)^. Revised guidelines later defined healthcare-associated infections (HAIs) as those occurring in hospitals, long-term care, ambulatory, and home settings^3)^. HAIs can harm healthcare workers, spread to others, and compromise care quality through understaffing^4)^. All healthcare professionals and students in related fields are expected to acquire knowledge and skills regarding standard precautions to prevent HAIs and protect frontline staff^5-16)^.

The Compliance with Standard Precautions Scale (CSPS), developed by Lam et al., is a validated 20- item self-administered tool used globally to assess compliance among nurses and students^17, 18)^. It has been translated into over 10 languages and adapted into clinical settings across a number of countries worldwide^19-28)^. The CSPS has been evaluated in multiple systematic reviews using COSMIN^29, 30)^ (the Consensus-Based Criteria for Selection of Health Measurement Instruments), a widely adopted framework for assessing health measurement tools. These reviews have shown that the CSPS demonstrates high psychometric quality in measuring adherence to standard precautions. Studies show that training improves self-reported compliance^22, 23, 26)^.

In Japan, standard precautions are incorporated into both undergraduate curricula and continuing professional development programs for healthcare workers. In facilities lacking infection control staff, community-based specialists support education^31)^. Despite frequent outbreaks and high occupational injury claims, compliance remains a challenge. For example, in Japan, 15,601 (65%) of 23,817 COVID-19- related occupational injury claims were submitted by healthcare workers^32)^. While several Japanese questionnaires have been developed to assess infection control practices, further validation is needed to ensure consistency and comparability with international findings^8, 10, 12)^. To address this, a well-established instrument should be adapted and validated for use in Japan.

Therefore, this study aimed to translate the CSPS into Japanese (CSPS-J) and validate it among Japanese nurses.

## Materials and Methods

### Study design and setting

**Figure 1:**
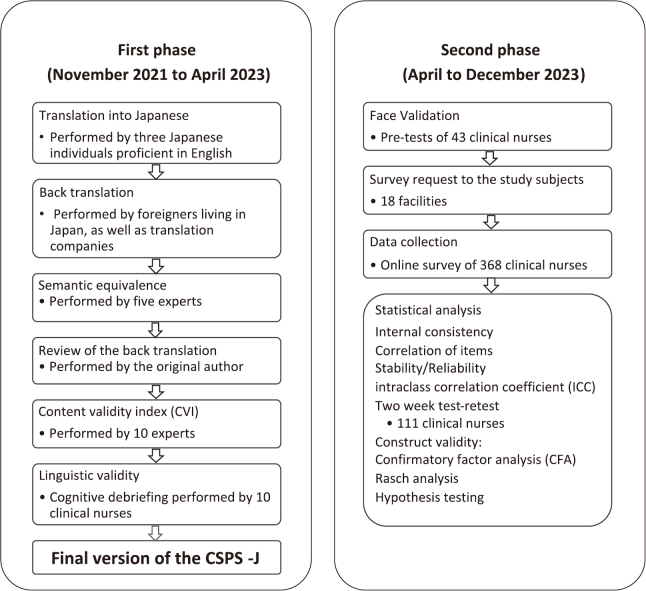
Flowchart of the study process

This methodological study included two phases (Figure 1). The first phase was to translate the original English scale into Japanese, by reviewing semantic equivalence, whereas phase two was to validate the translated survey in a group that represented the target population: clinical nurses from hospitals in the Kanto region of Japan. The survey was conducted between August and September 2023.

### Instrument

The 20 items of the CSPS comprise five dimensions: “use of protective equipment,” “disposal of sharps,” “disposal of waste,” “decontamination of spills and used articles,” and “prevention of cross-infection from person to person” (Table 1)^18)^. The participants were asked to respond regarding their actual infection control practice on a four-point adjective scale comprising: never (1), seldom (2), sometimes (3), or always (4). Four of the items (2, 4, 6, and 15) were negatively worded and therefore recoded accordingly. To reflect the expectation that healthcare professionals fully comply with standard precautions^33)^, a binary recoding was applied to the survey responses. For positively worded items, responses of “always” were coded as 1, and all other responses as 0. For negatively worded items, responses of “never” were coded as 1, and all others as 0. Self-reported compliance was calculated by summing these binary values, and defined as the proportion of respondents who selected “always” or “never” for all relevant items. A higher self-reported compliance indicated greater adherence to standard preventive measures. The total score (range, 0-20 points) was calculated as the sum of the scores for each of the 20 items.

The internal consistency (Cronbach’s alpha, 0.73), 2-week and 3-month test-retest reliability (Intraclass Correlation Coefficient; ICC, 0.74-0.79), construct validity (established using hypothesis testing and the known-group method), and criterion- related validity of the original version were satisfactory and have been previously reported^18)^.

**Table 1:** Dimensions and items (Q1-Q20) of the original compliance with standard precautions scale

Use of protective devices	
Q7	I remove Personal Protective Equipment (PPE) in a designated area.
Q10	I wear gloves when I am exposed to body fluids, blood products, and any excretion of patients.
Q13	I wear a surgical mask alone or in combination with goggles, face shield and apron whenever there is a possibility of a splash or splatter.
Q14	My mouth and nose are covered when I wear a mask.
Q15^†^	I reuse a surgical mask or disposable Personal Protective Equipment (PPE).
Q16	I wear a gown or apron when exposed to blood, body fluids or any patient excretions.
Disposal of sharps	
Q4^†^	I recap used needles after giving an injection.
Q5	I put used sharp articles into sharps boxes.
Q6^†^	The sharps box is disposed only when it is full.
Disposal of waste	
Q17	Waste contaminated with blood, body fluids, secretion and excretion is placed in red plastic bags irrespective of patient infection status.
Decontamination of spilled and used articles	
Q18	I decontaminate surfaces and equipment after use.
Q19	I wear gloves to decontaminate used equipment with visible soils.
Q20	I clean up spillage of blood or other body fluids immediately with disinfectants.
Prevention of cross-infection from person to person	
Q1	I wash my hands between patient contacts.
Q2^†^	I only use water for hand washing.
Q3	I use alcoholic hand rubs as an alternative if my hands are not visibly soiled.
Q8	I take a shower in case of extensive splashing even after I have put on Personal Protective Equipment (PPE).
Q9	I cover my wound(s) or lesion(s) with waterproof dressing before patient contacts.
Q11	I change gloves between patient contacts
Q12	I decontaminate my hands immediately after removal of gloves.

Responses were scored on a (1 to 4, respectively), excepting four items (†) scored from 4 (never) to 1 (always) that are counterintuitive to infection prevention.

### The first phase: translation of scales and review of semantic equivalence

The CSPS was translated by one of the authors (EU) and a Japanese expert with experience living abroad, assisted by non-Japanese medical interpreters and translation companies. The two translated scales were then adjusted and integrated by two other authors (YT, KT). The translated scales were reviewed for semantic equivalence in Japanese healthcare at a meeting of five infection control experts (AY, SI, KO, AS, and YB). Thereafter, several changes were made to the translated version. In question 1, “hand hygiene” was changed to “hand washing/hand sanitization”; for question 8, “use a shower” was changed to “use a shower or change clothes”; and in question 17, “red plastic bag” was changed to “infectious waste containers”. These changes were then verified by the CSPS developer.

The content validity of the CSPS-J was evaluated by a total of ten experts: five infection control expert authors (AY, SI, KO, AS, and YB), three infection control nurses, and two post-secondary teachers of nursing. Their clinical experience ranged 15-26 years (mean, 21.4 years). Content validity was assessed using a four-point Likert scale ranging 1 (not at all relevant) to 4 (very relevant) to rate each item for relevance to standard precautions. Content validity was calculated as item-level content validity ratings (I-CVI) and scale-level content validity ratings (S-CVI/Av). We considered ≥ 0.78 and ≥ 0.9 respectively to be acceptable I-CVI and S-CVI/Av values for 6-10 independent raters^34)^.

Linguistic validity of the CSPS was assessed through cognitive debriefing with ten nurses at a university hospital. Cognitive debriefing was conducted using a small group that was representative of the broader population who will use the scale^35, 36)^. After responding to the scale, interviews were conducted based on an interview guide evaluating explanatory text, question item content, choices, and difficulty understanding the language used. The participating nurses had an average of 12.9 years of clinical experience; their highest educational levels were junior college, university, or graduate school; and the wards they worked in were medical and surgical wards, paediatric wards, psychiatric wards, operating rooms, intensive care units, and outpatient wards. The final version of the CSPS-J (Appendix) was then considered complete after a final consultation with the original authors.

### The second phase: reliability and validity assessment

#### Web survey

Validation of the CSPS-J was conducted by outsourcing the online survey to a third-party research organization (Asahi Printing Industry. Co., Ltd., Maebashi, Gunma, Japan), which used the questionnaires consisting of the CSPS-J and questions regarding demographic variables (that is sex, age, license obtained, clinical experience, hospital ward, education, training status, and experience with infections on the job) to generate an online survey.

Before the online survey was established, a pre- test was conducted with 43 nurses recruited through snowball-sampling. This aspect focused on comprehensibility and feasibility^37)^. Face validity was assessed by checking the items of the CSPS-J, demographic characteristics, reporting the on-screen responses to the web survey, performing re-tests using coded entry, and reviewing response times, to confirm feasibility^37, 38)^. All items were answered in the pre-test, and its face validity was deemed adequate.

### Participants

The participants who responded to the online survey consisted of clinical nurses who provided direct care to patients at their facilities, aged 20-60 years. To enable broader applicability of the scale, the survey was conducted at multiple healthcare facilities located in both urban and regional areas, with varying bed capacities. The survey was conducted at facilities with consenting nursing administrators. We excluded nursing managers (such as directors and chief nurses) and part-time nursing staff who spent less time in direct clinical care. Our sample exceeded 200 participants, which is considered sufficient for validating the use of the scale across cultures with differing languages, lifestyles, and other contextual factors^37)^.

### Reliability and validity

Demographic variables from the data of 248 participants were analysed using cut-off points determined with reference to previous studies. Cut-off points for each variable were determined with reference to previous studies. Differences in self- reported compliance among nurses with and without infection prevention and control (IPC) training were tested using the Student’s *t*-test. Internal consistency was considered acceptable for Cronbach’s alpha values of ≥ 0.70. Stability was considered acceptable for ICC values ≥ 0.70 for the 2-week test-retest.

Construct validity was assessed by examining construct adequacy and response difficulty across items. Confirmatory factor analysis (CFA) was conducted using multiple fit indices, including the goodness-of-fit index (GFI), adjusted GFI (AGFI), comparative fit index (CFI), and Tucker-Lewis index (TLI), with values ≥ 0.90 considered satisfactory. Thresholds for the root mean square error of approximation (RMSEA) and standardized root mean square residual (SRMR) were set at ≤ 0.05, and statistical significance was defined as p < 0.05^39)^. Rasch analysis was also performed to evaluate item difficulty and model fit based on item response theory^40)^. Item difficulty was defined as ± 3 logits from the mean, whereas in-fit and out-fit statistics were interpreted as adequate (0.5-1.5) or acceptable (1.5-2.0). Unidimensionality was supported by an eigenvalue < 2^41)^. The hypothesis that nurses with more experience would show higher compliance was tested to examine construct validity, based on consistent findings in the literature^22, 23, 26)^. Differences in self-reported compliance between nurses with and without IPC training were tested using the Student’s *t*-test.

The statistical packages used included IBM Amos (IBM: New York, NY, USA) version 24.0 for CFA, WINSTEPS (Winsteps.com, Beaverton, OR, USA) version 5.3.3 for Rasch analysis, and IBM SPSS (IBM: New York, NY, USA) version 24.0 for all other analyses.

### Ethical considerations

This study was approved by the ethical committee of our institution (approval no.: E22-0272-M03; issued May 1, 2023). Permission to translate the CSPS was granted by the developer (Lam, SC). The explanatory document issued to the study participants included an explanation and consent form regarding the purpose and procedures of the study, anonymity of data collection, handling of data, and conflicts of interest.

Informed consent was obtained through both oral affirmation and written consent prior to the interview for the experts and cognitive debriefing participants. For the online survey, consent was obtained through a specific form, and a code was used to identify participants, while maintaining anonymity when test-retests were conducted.

## Results

The analysis was conducted using response data from 248 nurses across 18 healthcare facilities (demographic characteristics presented in Table 2). The wards the nurses worked in included internal and surgical, recovery and rehabilitation, long-term care, psychiatric, obstetrics and paediatrics, outpatient, acute-care emergency, operating rooms, and infectious disease wards. A total of 189 (76.2%) of the nurses had received prior training regarding standard infection-related precautions. The prior hospital infection exposure experience included droplet infection training, in 140 (56.5%) of the participants, and needle-stick and other percutaneous and transmucosal exposure training, in 67 (27.0%) participants.

Table 3 presents the item- and dimension-level fit rates for the CSPS-J based on responses from 248 participants. The overall self-reported compliance rate was 63.3%. Self-reported compliance by dimension were low for “disposal of sharps” and “prevention of cross-infection from person to person.”

Table 4 shows the reliability and validity results of the CSPS-J in the participant nurses. Its content validity values were within acceptable ranges, with all I-CVI scores exceeding 0.80 and the S-CVI/Av reaching 0.96. Linguistic validity was examined through cognitive debriefing sessions lasting 30-90 min per participant (mean: 57 min). Based on feedback, five items identified as difficult to understand were revised by adjusting word order and terminology prior to the web-based survey. Internal consistency and ICC values were acceptable. CFA showed overall good model fit. Rasch analysis further supported the validity of the scale, with item difficulty, in-fit, and out-fit statistics falling within acceptable ranges, and unidimensionality confirmed. We conducted hypothesis testing to explore whether nurses with IPC training showed higher compliance with standard precautions. The results indicated that self-reported compliance of the nurses who had previously participated in training was significantly higher than that of the nurses who had never participated (n = 248: t = 2.06 p = 0.042).

**Table 2:** Demographic characteristics of respondent nurses (n = 248) variables

Variables		Frequency (%) (n=248)
Sex	Male	27 (10.9)
	Female	217 (87.5)
	Other/no answer	4 (1.6)
Age, years	< 30	88 (35.5)
	≥ 30	160 (64.5)
License (multiple answers permitted)	Registered nurse	241 (97.2)
	Public health nurse	9 (3.6)
	Midwives	11 (4.4)
Clinical experience, years	< 5	50 (20.2)
	≥ 5	198 (79.8)
Hospital beds (beds)	< 300	71 (28.6)
	≥ 300	177 (71.4)
Education (highest degree)	Graduate school/College/University	75 (30.2)
	Junior college/Professional training college	173 (69.8)
Experience with training courses	Yes	189 (76.2)
	No	59 (23.8)
Experience with infections on the job		
Experience with contact infections (e.g., MRSA and other drug-resistant bacteria, viral gastroenteritis, scabies, etc.)	Yes	59 (23.8)
	No	189 (76.2)
Experience with droplet infections (e.g., COVID-19, influenza, mycoplasma, rubella or others)	Yes	140 (56.5)
	No	108 (43.5)
Experience with airborne infections (e.g., tuberculosis, chickenpox, measles or others)	Yes	47 (19.0)
	No	201 (81.0)
Experience with percutaneous and transmucosal exposure (e.g., needlesticks, cut, skin/mucosa contamination)	Yes	67 (27.0)
	No	181 (73.0)

COVID-19 = coronavirus disease; MRSA = methicillin-resistant *Staphylococcus aureus*

**Table 3:** Compliance with the dimensions and items of the CSPS-J among responding nurses (n = 248)

CSPS Japanese version items^a^				frequency (%)				Self-reported compliance (%)	Binary recoding (SD)
				Never	Seldom	Sometimes	Always		
Use of protective devices									
	Q7	I remove Personal Protective Equipment (PPE) in a designated area.		4 (1.6)	17 (6.9)	66 (26.6)	161 (64.9)	64.9	
	Q10	I wear gloves when coming into contact with blood, bodily fluids, secretions, and any other excretions from patients.		0 (0.0)	0 (0.0)	27 (10.9)	221 (89.1)	89.1	
	Q13	I wear a surgical mask alone or a combination of a surgical mask, goggles, face shield, and apron whenever there is a possibility of splashing.		1 (0.4)	3 (1.2)	78 (31.5)	166 (66.9)	66.9	
	Q14	When I wear a mask, my mouth and nose are covered with the mask.		0 (0.0)	0 (0.0)	9 (3.6)	239 (96.4)	96.4	
	Q15^†^	I reuse surgical masks and disposable Personal Protective Equipment (PPE).		175 (70.6)	31 (12.5)	23 (9.3)	19 (7.7)	70.6	
	Q16	I wear a gown or an apron when exposed to blood, bodily fluids, secretions, or any other excretions of patients.		0 (0.0)	7 (2.8)	92 (37.1)	149 (60.1)	60.1	
		average overall compliance for dimension						74.7	4.5 (134)
Disposal of sharps									
	Q4^†^	I recap used needles after giving an injection.		134 (54.0)	74 (29.8)	34 (13.7)	6 (2.4)	54.0	
	Q5	I put used sharp objects into a sharps disposal containers.		2 (0.8)		14 (5.6)	232 (93.5)	93.5	
	Q6^†^	I dispose of the sharps disposal container only when it is full.		37 (14.9)	74 (29.8)	91 (36.7)	46 (18.5)	14.9	
		average overall compliance for dimension						54.1	1.6 (0.68)
Disposal of waste									
	Q17	I place garbage that has been contaminated with blood, bodily fluids, secretions, and any other excretions in an infectious waste containers, regardless of patient infection status.		7 (2.8)	7 (2.8)	34 (13.7)	200 (80.6)	80.6	
		average overall compliance for dimension						80.6	0.8 (0.40)
Decontamination of spilled and used articles									
	Q18	I decontaminate environmental surfaces and utensils after use.		3 (1.2)	10 (4.0)	118 (47.6)	117 (47.2)	47.2	
	Q19	I wear gloves when disinfecting used equipment that is visibly soiled.		6 (2.4)	9 (3.6)	24 (9.7)	209 (84.3)	84.3	
	Q20	I wipe up and sanitize spilled blood and other bodily fluids immediately.		1 (0.4)	3 (1.2)	33 (13.3)	211 (85.1)	85.1	
		average overall compliance for dimension						72.2	2.2 (0.84)
Prevention of cross-infection from person to person									
	Q1	I perform hand hygiene (hand washing/hand sanitization) every time I come in contact with patients.		1 (0.4)	3 (1.2)	93 (37.5)	151 (60.9)	60.9	
	Q2^†^	I wash my hands with water only.		198 (79.8)	39 (15.7)	9 (3.6)	2 (0.8)	79.8	
	Q3	I use an alcohol-based hand rub instead of soap and water when my hands are not visibly dirty.		11 (4.4)	23 (9.3)	143 (57.7)	71 (28.6)	28.6	
	Q8	I use a shower or change clothes when there is a concern of extensive splashing even after wearing Personal Protective Equipment (PPE).		57 (23.0)	73 (29.4)	79 (31.9)	39 (15.7)	15.7	
	Q9	I cover my wound(s) and/or skin lesion(s) with a waterproof dressing before coming into contact with a patient.		25 (10.1)	44 (17.7)	98 (39.5)	81 (32.7)	32.7	
	Q11	I change gloves every time I come into contact with a patient.			5 (2.0)	50 (20.2)	193 (77.8)	77.8	
	Q12	I perform hand hygiene immediately after removing my gloves.			5 (2.0)	88 (35.5)	155 (62.5)	62.5	
		average overall compliance for dimension						51.1	3.5 (1.60)
		Overall CSPS-J						63.3	12.66 (3.50)

^a^ The items are in Japanese, but their English translations are shown here. Responses were scored on a progressive Likert scale, from 1 to 4, except for four items (†) that were scored on a regressive Likert scale, from 4 (never) to 1 (always). CSPS-J = Compliance with Standard Precautions Scale Japanese version; SD = standard deviation

**Table 4:** Reliability and validity of the CSPS-J assessed using data from 248 nurses

Measure	Methods	Result
Reliability		
Internal Consistency	Cronbach’s α	0.73
Stability	Two-week post-test-retest reliability, Intraclass correlation coefficient (ICC)	0.97, *p* < 0.001 (n = 104)
Validity		
Face validity	Reviewer by target population^a^	Interpreted and answerable
Linguistic validity	Cognitive debriefing	5-item adjustment
Content validity	Content validity index (CVI)	S-CVI = 0.96; I-CVI = 0.80-1.00
Structural validity	Confirmatory factor analysis (CFA)	χ^2^ = 203.243 *p* = 0.042 GFI = 0.927, AGFI = 0.910, CFI = 0.928, TLI = 0.920, RMSEA = 0.028, SRMR = 0.053
	Rasch analysis	-2.58 to 2.13 logits in-fit MNSQ = 0.60-1.97 out-fit MNSQ = 0.56-1.97 Unidimensional eigenvalue = 1.85
Hypothesis testing	Training vs. no training	With: mean = 12.95, SD = 3.23 Without: mean = 11.73, SD = 4.16 t = 2.06, *p* = 0.042

^a^ Results calculated based on data from 43 nurses. CSPS-J = Compliance with Standard Precautions Scale Japanese version; SD = standard deviation. S-CVI = scale-level content validity index; I-CVI = item-level content validity index.

## Discussion

This study was conducted in two standardized phases to ensure equivalence between the original English and Japanese versions of the CSPS and comprehensively examine its psychometric properties. Our results support the satisfactory reliability and validity of the CSPS-J. To date, Japan lacks a reliable and valid measure of nurses’ compliance with standard precautions. Therefore, we believe that comprehensive and objective evaluations of the CSPS-J will contribute to improving the measurement of infection prevention and control (IPC) practices among nurses in clinical settings.

The semantic equivalence of the CSPS-J has been confirmed several times by experts in infection prevention and by the original authors. The original English version was reviewed by experts and adapted to the Japanese medical context, preserving the quality of the scale while enhancing usability for nurses. In addition to its methodological strengths, the CSPS-J has practical implications. Specifically, it may facilitate the visualization of educational and training effects related to standard precautions, support self-assessment and quality improvement within and across healthcare institutions, and promote international comparative research using standardized tools.

The CSPS-J showed satisfactory internal consistency, comparable to that of the original version^24)^ (Cronbach’s alpha = 0.73). The 2-week test-retest yielded an ICC of 0.97 (p < 0.001), which was higher than the ICC of 0.79 (p < 0.01) reported for the original version^18)^, indicating high stability in measuring compliance with standard precautions among Japanese nurses.

The validity of the CSPS-J was confirmed using multiple methods. Linguistic validity was supported through cognitive debriefing with clinical nurses, during which five specific items were examined. Cognitive debriefing is recommended when adapting scales across cultures^37, 38)^, and the correct interpretation of CSPS-J items may help Japanese nurses to self-assess their infection prevention competencies. The construct validity was supported by CFA, with the CSPS-J demonstrating better goodness- of-fit indices than the Italian version^20)^ (χ^2^ = 508.26, p < 0.001, CFI = 0.90, TLI = 0.87, and RMSEA = 0.09). Rasch analysis further confirmed acceptable goodness-of-fit and unidimensionality, similar to the findings of the Turkish version^28)^. These results suggest that Japanese nurses interpreted and answered the items confidently.

Consistent with previous studies^22, 23, 26)^, self- reported compliance was positively associated with clinical training experience. This pattern has been observed across different cultures and contexts, supporting its appropriateness as an indicator of construct validity. These findings highlight the importance and effectiveness of training in reducing healthcare-associated infections.

In this study, the CSPS-J was validated using a representative sample of the intended population: clinical nurses working in various hospital wards. Thus, the tool has the potential to be widely used across Japan and can be tailored to meet the needs of different facilities. The characteristics of the respondents also enabled us to examine the impact of training on compliance. Future studies should consider survey methodologies that allow participants to provide detailed background information.

## Limitations

This study collected responses from various facilities to minimize respondent bias. However, the participating nurses may have been influenced by factors such as the facility type or years of experience. As this study focused solely on nurses, caution is required when generalizing the findings to other healthcare professionals. Future studies should examine the applicability of the CSPS-J to other healthcare professionals and their trainees.

Compliance with standard precautions was assessed through self-report, which may be subject to social desirability bias. In addition, the binary scoring system has advantages, such as facilitating international comparisons and emphasizing full compliance, but it may also reduce sensitivity to incremental behavioral changes.

Future research should identify the factors influencing compliance, such as motivation and risk awareness, and explore ways to improve it. To further strengthen the validity of the CSPS-J, observational methods and their correlations with related measures should be examined. More rigorous evaluations of translation equivalence, such as parallel translation with concordance ratings and cross- linguistic comparisons^42)^, may be required because semantic equivalence alone may be insufficient. Finally, the CSPS-J can be applied in future studies involving other healthcare professionals or used to evaluate educational interventions.

## Conclusion

This two-phase methodological study adapted the English CSPS into the CSPS-J for nurses in Japanese clinical settings. The results showed that the CSPS-J is equivalent to the original CSPS, with satisfactory reliability and validity. This tool is therefore ready to use for measuring compliance with infection control practices among Japanese nurses.

## Author contributions

KT: conceptualization, data curation, formal analysis, funding acquisition, investigation, methodology, translated the scales, scale harmonization and integration, project administration, resources, visualization, writing - original draft, writing - review & editing; HI: conceptualization, methodology, project administration, visualization, writing - review & editing; AY: conceptualization, investigation, cultural adaptation, writing - review & editing; SL: conceptualization, methodology, visualization, writing - review & editing; SI: cultural adaptation, writing - review & editing; KO: cultural adaptation, writing - review & editing; AS: cultural adaptation, writing - review & editing; YB: cultural adaptation, writing - review & editing; YT: translated the scales, scale harmonization and integration, visualization, writing - review & editing; EU: translated the scales, scale harmonization and integration, writing - review & editing; KY: conceptualization, methodology, project administration, visualization, writing - review & editing, supervision. All authors listed meet the authorship criteria and agreed to the content of the manuscript.

## Conflicts of interest statement

The authors declare that there are no conflicts of interest.

## Supplementary Material

**Appendix:** CSPS (CSPS-J) Subscales and items: original English, Japanese translation, and back translation

Original English version		Japanese translation (CSPS-J)	Back translation (English)
Use of protective devices		防護具の使用	Use of protective devices
Q7	I remove Personal Protective Equipment (PPE) in a designated area.	私は指定の場所で個人防護具（PPE）を外す．	I remove Personal Protective Equipment (PPE) in a designated area.
Q10	I wear gloves when I am exposed to body fluids, blood products, and any excretion of patients.	私は患者の血液，体液，分泌物，あらゆる排泄物に触れるときは手袋を着用する．	I wear gloves when coming into contact with blood, bodily fluids, secretions, and any other excretions from patients.
Q13	I wear a surgical mask alone or in combination with goggles, face shield and apron whenever there is a possibility of a splash or splatter.	私は飛沫が飛び散る可能性がある場合はいつでも，サージカルマスクのみあるいはサージカルマスクとゴーグル，フェイスシールド，エプロンを組み合わせて着用する．	I wear a surgical mask alone or a combination of a surgical mask, goggles, face shield, and apron whenever there is a possibility of splashing.
Q14	My mouth and nose are covered when I wear a mask.	私はマスクを着用するときは口と鼻はマスクで覆われている．	When I wear a mask, my mouth and nose are covered with the mask.
Q15^†^	I reuse a surgical mask or disposable Personal Protective Equipment (PPE).	私はサージカルマスクや使い捨ての個人防護具（PPE）を再利用する．	I reuse surgical masks and disposable Personal Protective Equipment (PPE).
Q16	I wear a gown or apron when exposed to blood, body fluids or any patient excretions.	私は患者の血液，体液，分泌物，あらゆる排泄物に触れるときはガウンまたはエプロンを着用する．	I wear a gown or an apron when exposed to blood, bodily fluids, secretions, or any other excretions of patients.
Disposal of sharps		鋭利な物品の処理	Disposal of sharps
Q4^†^	I recap used needles after giving an injection.	私は注射後に使用済みの針にリキャップする．	I recap used needles after giving an injection.
Q5	I put used sharp articles into sharps boxes.	私は使用済みの鋭利な物を針捨てボックスに入れる．	I put used sharp objects into a sharps disposal containers.
Q6^†^	The sharps box is disposed only when it is full.	私は針捨てボックスがいっぱいになったときだけ処分する．	I dispose of the sharps disposal container only when it is full.
Disposal of waste		廃棄物の処理	Disposal of waste
Q17	Waste contaminated with blood, body fluids, secretion and excretion is placed in red plastic bags irrespective of patient infection status.	私は血液，体液，分泌物，あらゆる排泄物で汚染された廃棄物は患者の感染状況に関わらず感染性廃棄物容器に入れる．	I place garbage that has been contaminated with blood, bodily fluids, secretions, and any other excretions in an infectious waste containers, regardless of patient infection status.
Decontamination of spilled and used articles		漏出物や使用済み物品の汚染除去	Decontamination of spilled and used articles
Q18	I decontaminate surfaces and equipment after use.	私は，使用後の環境表面や器具を消毒する．	I decontaminate environmental surfaces and utensils after use.
Q19	I wear gloves to decontaminate used equipment with visible soils.	私は目に見える汚れが付いた使用済みの器具を消毒する際は手袋を着用する．	I wear gloves when disinfecting used equipment that is visibly soiled.
Q20	I clean up spillage of blood or other body fluids immediately with disinfectants.	私は血液やその他の体液がこぼれた場合は直ちに拭き取り消毒する．	I wipe up and sanitize spilled blood and other bodily fluids immediately.
Prevention of cross-infection from person to person		人から人への交差感染の防止	Prevention of cross-infection from person to person
Q1	I wash my hands between patient contacts.	私は患者に接触するたびに手指衛生（手洗い・手指消毒）を行う．	I perform hand hygiene (hand washing/hand sanitization) every time I come in contact with patients.
Q2^†^	I only use water for hand washing.	私は手洗いは水だけで行う．	I wash my hands with water only.
Q3	I use alcoholic hand rubs as an alternative if my hands are not visibly soiled.	私は目に見える汚れが手に無い場合は石けんと流水の代わりに擦式アルコール手指消毒液を使う．	I use an alcohol-based hand rub instead of soap and water when my hands are not visibly dirty.
Q8	I take a shower in case of extensive splashing even after I have put on Personal Protective Equipment (PPE).	私は個人防護具（PPE）を着用していた後でも広範囲に飛散している恐れがある場合はシャワーを使う，または更衣する．	I use a shower or change clothes when there is a concern of extensive splashing even after wearing Personal Protective Equipment (PPE).
Q9	I cover my wound(s) or lesion(s) with water-proof dressing before patient contacts.	私は患者に接する前に自分の創傷や皮膚の病変を防水性ドレッシング材で覆う．	I cover my wound(s) and/or skin lesion(s) with a waterproof dressing before coming into contact with a patient.
Q11	I change gloves between patient contacts	私は患者に接触するたびに手袋を交換する．	I change gloves every time I come into contact with a patient.
Q12	I decontaminate my hands immediately after removal of gloves.	私は手袋を外したあとは直ちに手指衛生を行う．	I perform hand hygiene immediately after removing my gloves.
Likert scale never (1), seldom (2), sometimes (3), always (4)		リッカート尺度 １=全く当てはまらない，２=ほとんど当てはまらない，３=時々当てはまる，４=常に当てはまる	Likert scale never (1), seldom (2), sometimes (3), always (4)

Responses were scored on a progressive Likert scale, from 1 to 4, except for four items (†) that were scored on a regressive Likert scale, from 4 (never) to 1 (always).
